# Dose–Response Relationship between Physical Workload and Specific Shoulder Diseases—A Systematic Review with Meta-Analysis

**DOI:** 10.3390/ijerph17041243

**Published:** 2020-02-14

**Authors:** Andreas Seidler, Karla Romero Starke, Alice Freiberg, Janice Hegewald, Albert Nienhaus, Ulrich Bolm-Audorff

**Affiliations:** 1 Institute and Policlinic of Occupational and Social Medicine (IPAS), Faculty of Medicine Carl Gustav Carus, Technische Universität Dresden, 01307 Dresden, Germany; karla.romero_starke@tu-dresden.de (K.R.S.); alice.freiberg@tu-dresden.de (A.F.); janice.hegewald@tu-dresden.de (J.H.); 2 Department of Occupational Medicine, Public Health and Hazardous Substances, Statutory Accident Insurance and Prevention in the Health and Welfare Services, 22089 Hamburg, Germany; Albert.Nienhaus@bgw-online.de; 3 Competence Centre for Epidemiology and Health Services Research for Healthcare Professionals (CVcare), University Medical Centre Hamburg-Eppendorf (UKE), 20246 Hamburg, Germany; 4 Division of Occupational Health, Department of Occupational Safety and Environment, Regional Government of South Hesse, 65197 Wiesbaden, Germany; ulrich.bolm-audorff@rpda.hessen.de; 5 Institute and Outpatient Clinic for Occupational and Social Medicine, University Medical Center Gießen, Justus-Liebig-University, 35392 Gießen, Germany

**Keywords:** rotator cuff lesions, physical workload, dose-response relationship, doubling dose, musculoskeletal diseases of the shoulder, occupational disease

## Abstract

Several epidemiological studies have found an association between shoulder-loaded work activities and specific shoulder diseases. No study has derived the dose-response relationship and resulting doubling dose, important for the recognition of occupational diseases. This systematic review is an update of the van der Molen et al. (2017) review. Based on its methodologies, we identified new studies published up to November 2018. The dose-response relationship between physical occupational demands (hands at/above shoulder level, repetitive movements, forceful work, hand-arm vibrations) and specific shoulder diseases (defined as ICD-10 M 75.1-5: rotator cuff syndrome, bicipital tendinitis, calcific tendinitis, impingement, and bursitis) was derived. No evidence for sex-specific differences in the dose-response relationship was found. If there were at least two studies with comparable exposures, a meta-analysis was carried out. The pooled analysis resulted in a 21% risk increase (95% CI 4–41%) per 1000 h of work with hands above shoulder level. A meta-analysis was not possible for other occupational burdens due to the low number of studies and differing exposure measurements; an estimate of the doubling dose was made based on the cohort study of Dalbøge et al. (2014). To conclude, the present systematic review with meta-analysis contributes to knowledge of the level of exposure at which specific shoulder diseases—particularly rotator cuff lesions—should be recognized as an occupational disease.

## 1. Introduction

Several epidemiological studies [1,2,3,4,5,6,7] have shown a positive association between shoulder-loaded occupational activities and specific shoulder diseases, especially with respect to rotator cuff lesions (including lesions of the supraspinatus tendon) [3,6]. For a systematic review of the evidence, see the review by van der Molen et al. 2017 [8]. Accordingly, the Medical Advisory Board on Occupational Diseases in Germany is currently discussing the topic “musculoskeletal diseases of the shoulder (rotator cuff lesion)” (for more information see https://www.bmas.de/DE/Themen/Soziale-Sicherung/Gesetzliche-Unfallversicherung/der-aerztliche-sachverstaendigenbeirat-berufskrankheiten.html).

At the international level, the doubling of the risk of disease plays an important role in the introduction and recognition of an occupational disease, because doubling risk is often “translated” into a probability of causation of 50% [9]. In Germany, in the case of newer occupational diseases, the legal definition often specifies the dose limit based on epidemiological studies investigating the doubling dose. Thus, the doubling dose is of regulatory, as well as preventive medical importance with respect to shoulder diseases. There is a research gap in this regard: there has been no study as of yet that deals with the concrete derivation of a dose-response relationship for specific shoulder diseases. Furthermore, there is only little research on the differences in the risks of women and men. This systematic review intends to help close these research gaps.

The aim of this systematic review is to derive the dose-response relationship between physical occupational loads (1. working with the hands at or above shoulder level, 2. working with repetitive movement of the upper arm at the shoulder joint, 3. forceful shoulder exertions, and 4. working with vibration of the hands and arms) and specific shoulder diseases. Just as van der Molen et al. [8], we equated the term “specific shoulder disease” with “subacromial pain syndrome”, defined as “all non-traumatic, usually unilateral, shoulder problems that cause pain, localised around the acromion, often worsening during or subsequent to lifting of the arm”. In accordance with van der Molen et al. [8], the following ICD-10 diagnoses were subsumed under the subacromial pain syndrome: rotator cuff syndrome, including tendinitis of the supraspinatus, infraspinatus and/or non-traumatic tears and ruptures (ICD-10 code M75.1); bicipital tendinitis (M75.2); calcific tendinitis (M75.3); impingement (M75.4); and bursitis (M75.5).

In our systematic review, the dose-response relationship for specific shoulder diseases is derived. Specifically, the dose-response relationship between physical workload and lesions of the shoulder rotator cuff is addressed. Particular attention is paid to the question of whether there is evidence of different disease risks for men and women.

## 2. Materials and Methods

The methodological approach in this paper was based on the systematic review published in 2017 by van der Molen et al. [8]. The same approach was used for the inclusion and exclusion criteria, the systematic literature search, the title-abstract and full-text review, as well as for the quality assessment of the included studies. In this respect, the present work can be understood to be an update of the aforementioned systematic review. Methodological extensions of the present work are due to:

1. the aim of deriving a dose-response relationship
2. the investigation of the risk for rotator cuff syndrome, in addition to the original investigation of the risks for “specific shoulder diseases” by van der Molen and colleagues [8]
3. a sex-specific analysis to estimate and compare risks for women and men.

### 2.1. Criteria for Study Inclusion and Electronic Search

**Figure 1:**
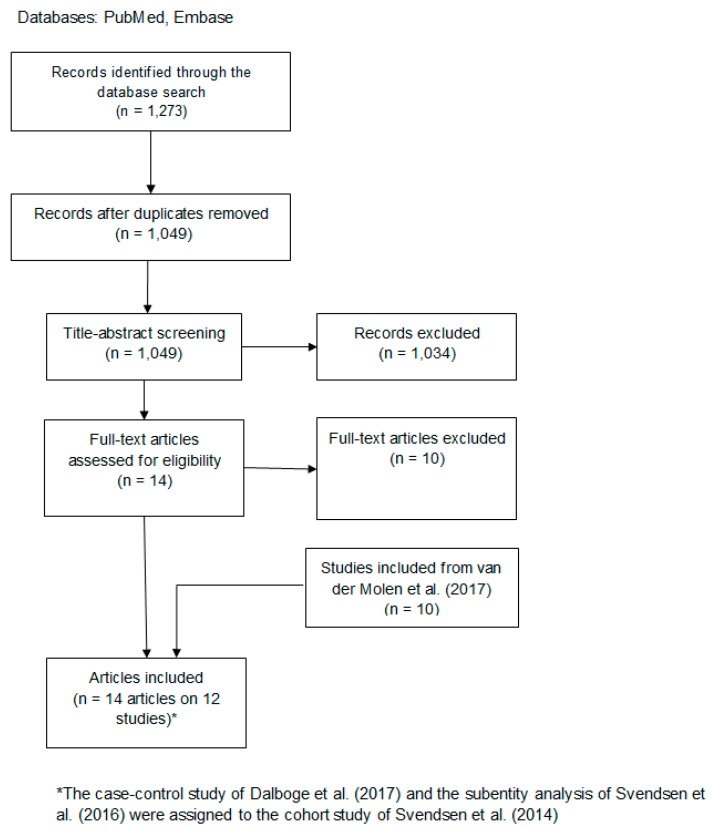
PRISMA Flow Diagram.

The same inclusion criteria as that of the 2017 systematic review from van der Molen and colleagues [8] was used. The 10 studies included in the aforementioned systematic review were initially included [2,3,4,6,7,10,11,12,13,14]. In addition, we carried out an electronic search from January 2016 to 16 November 2018 using the Pubmed and Embase search strings used in the previous review [8] (see PRISMA flowchart in Figure 1). The Pubmed search resulted in 509 hits, and the Embase search resulted in 764 hits. After clearing duplicates, 1049 hits remained.

### 2.2. Title-Abstract and Full-Text Screening

Two scientists (A.S., U.B.-A.) independently reviewed the 1049 abstracts. In case of differing assessments, consensus was reached between the parties. Fifteen texts were included in the full-text screening. The full texts were again reviewed independently by two scientists (A.S., U.B.-A.). Differing decisions were resolved by consensus. Four publications were included after the full-text review [15,16,17,18]. The reasons for exclusion of certain publications are shown in Supplementary Materials, Table S1. The two publications by Dalbøge and colleagues were based on a study [7] that had already been included in the 2017 review by van der Molen and colleagues [8]. Both studies contain amplified analyses [15,16], and one is a nested case-control study from the original cohort [16]. Since both publications from Møller and colleagues [17] and Thygesen and colleagues [18] were based on the same study (The Copenhagen Airport Study), they qualified as one study in our update.

### 2.3. Data Extraction and Quality Assessment

The extraction of the study characteristics and quality assessment was analogous to the previous review [8]. For extraction of the study characteristics, see Table 1; for quality assessment of the new publications included in this review, see Table 2; and for studies included in the previous review, see [8]. There was no new quality assessment of the studies that had been included in the review by van der Molen and colleagues.

The extraction of the study results was done both for studies published from 2016 and those published in the van der Molen et al. 2017 review. The study results were extracted separately for the examined exposures: (1) working with the hands at or above shoulder level, (2) working with repetitive movement of the upper arm at the shoulder joint, (3) forceful shoulder exertions, and (4) working with vibration of the hands and arms.

### 2.4. Synthesis of Evidence and Statistical Analysis

If a minimum of three studies with comparable exposure assessments were found for one of the above-mentioned four exposures, the studies were summarized in a meta-analysis using a two-stage procedure, similar to the analysis performed by Ijaz and colleagues [19]. First, a dose-response curve was estimated for the individual studies using regression analysis [20]. The risk estimates given by the study authors were assigned to the median of lower and upper category limits. When upper categories were open ended, we used the width of the adjacent category to calculate an upper or lower category limit (see [21]). Insofar as the median (or arithmetic mean values) of the exposure categories were given by the study authors, the respective risk estimators were assigned to them. In our own case control study [3], the course of the dose-response relationship was recalculated on the basis of continuous exposure data instead of the published categorized data. The dose-response curves of the individual studies were then combined into a pooled risk estimate of the dose-response relationship using random-effects meta-analysis. Based on this pooled risk estimate, the doubling dose was calculated as the exposure that corresponds to the relative risk estimate of two. If a minimum of three studies with comparable exposure assessments were not available for any of the four exposures, then a risk ratio across the categories was calculated for individual studies using a variance-weighted linear model, and from this, the doubling dose was estimated. For continuous data, linear, squared, and cubic models were derived and their fit compared using the Akaike Information Criterion (AIC). In general, the model with the lowest AIC value was chosen, but if the AIC vales were similar, the simplest model was taken. The risk estimates between men and women were compared descriptively. There was no update on the quality of evidence using the Grading of Recommendations Assessment, Development, and Evaluation (GRADE) approach [22], used in the previous review by van der Molen [8].

## 3. Results

The extraction of the characteristics of the new studies, including extensions to the 2004 Dalbøge and colleagues study [7], can be found in Table 1. The quality assessment of the new studies, including the 2017 nested case-control study from Dalbøge and colleagues [16] can be found in Table 2.

Van der Molen [8] created a total score from 16 quality criteria; studies were considered to be of high quality if they met at least 11 out of 16 criteria. According to this evaluation scheme, all new studies are of high quality: three studies received the highest score of 16; the 2017 nested case-control study by Dalbøge [16] achieved a score of 14 because the response was below 70% (60.1%) and because only little information was given about non-responders. Nevertheless, the results of this nested case-control study [16] have an advantage over the results of the studies on which it was based [7,23] because the exposure estimates in the nested case-control study are based on measurement-based values. The results of the cohort study mentioned above are taken into account in the context of sensitivity analyses. The study by Svendsen and colleagues (2004b) [6] examines a subset of the study by Svendsen (2004a) [4] with magnetic resonance imaging (MRI); based on the MRI-supported diagnosis, the Svendsen subset study (2004b) [6] is preferred to the main Svendsen study (2004a) [4]. The risk estimates of all studies (including the older studies identified by the previous van der Molen review [8] which enable a quantification of the cumulative exposure duration) are given in Table 3, Table 4, Table 5 and Table 6.

### 3.1. Working with the Hands at or Above Shoulder Level

#### 3.1.1. Cumulative Exposure Calculation and Risk of Disease

Three studies provided quantitative information on the duration of arm lifting at or above shoulder level, from which the cumulative duration of the corresponding activities can be estimated (Table 3). The Danish cohort study (comprising [7,15,23]), and its resulting nested case-control study [16], only give the cumulative duration of arm lifting over the past 10 years. If an average age of 20 years is assumed for entry into physically demanding work, an average duration of the activity (for the sick person up to the operation) of 30 to 36 years can be estimated. To take this into account, the cumulative duration of arm lifting was multiplied by a correction factor of 3, 3.4 or 3.6 (see Table 3). To give an example for the calculation of the cumulative lifetime hours of arm elevation in the highest exposure category of Dalbøge et al. 2014 [7], the correction factor 3 (to extrapolate from the observed 10-year timeframe) was multiplied by the median exposure parameter of 15 arm elevation years in this category and by the average daily exposure of 0.5 h underlying 1 arm elevation year and by 220 working days per year, resulting in 4950 cumulative lifetime hours.

**Figure 2:**
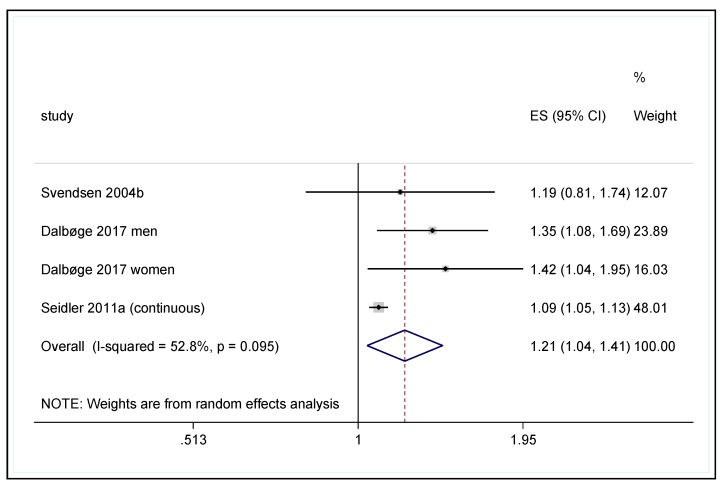
Forest plot of specific shoulder disease risk due to above-shoulder level work.

In the meta-analytical “core analysis”, the results of the Danish nested case control study [16], the MRI-supported cross-sectional study by Svendsen and colleagues [6], and our own case-control study [3] with continuous data were pooled together. The result of the core analysis shows a pooled risk estimate of OR 1.21 (95% CI 1.04–1.41) per 1000 h of work above shoulder level (Figure 2). Based on this result, the “best estimate” of the doubling dose is 3636 h of work with hands on or above the shoulder level.

Several sensitivity analyses were performed in addition to the core analysis. First, the original categorized data from Seidler and colleagues [3] was used instead of the continuous data. The pooled risk estimate was very similar to the estimates in the core analysis (OR = 1.20, 95% CI 1.09–1.33), corresponding to a doubling dose of 3802 h. In a second sensitivity analysis, the Danish cohort study [7] was included in the meta-analysis instead of the Danish nested case control study [16]. As a result, there was a slight reduction in the pooled risk estimate (OR = 1.15; 95% CI 1.04–1.26), and therefore there was an increase in doubling dose to 4959 h. It should be kept in mind that the Danish study (both the core analysis and the nested case control study) refer not only to rotator cuff lesions, but to a significantly broader spectrum of shoulder diseases treated with surgery. Therefore, in a third sensitivity analysis, the data from the Danish cohort study [7] (included in the second sensitivity analysis) were limited to only cases with a diagnosis of a rotator cuff lesion [23]. Compared with the second sensitivity analysis, this resulted in a slightly higher OR (OR = 1.16; 95% CI 1.03–1.30) and a doubling dose of 4670 h. A summary of the sensitivity analyses along with their forest plots is presented in Supplementary Table S2 and Supplementary Figures S1–S3.

#### 3.1.2. Sex-Specific Differences in Disease Risk

Men and women categories were only included in the Danish cohort study [7] and in the corresponding nested case-control study [16]. Dalbøge and colleagues [7] found comparable risk progressions for men and women and the sex-specific interaction term did not reach statistical significance. The nested case-control study found a slightly higher risk estimate for slightly higher loads in the highest exposure category for men. Overall, comparable dose-response curves for work at or above shoulder level can be assumed for men and women.

#### 3.1.3. Results of Other Studies

In the cohort study by Bodin and colleagues (2012, no table) [10], the risk of a rotator cuff syndrome due to arm being raised above shoulder level in a time span of at least two hours per shift is higher for men than for women. However, neither the exact duration of the daily exposure nor the number of exposed shifts were taken into account in the aforementioned study. This also applies to the cross-sectional study by Miranda and colleagues (2005, no table) [12]: the risk of rotator cuff syndrome is comparable for men and women, and is dependent on the number of working years in which they worked with the hand over the shoulder daily for at least one hour.

### 3.2. Working with Repetitive Movement of the Upper Arm at the Shoulder Joint

#### 3.2.1. Cumulative Exposure Calculation and Risk of Disease

Only the Danish cohort study [7,15] and the nested case-control study [16] allowed for a quantitative analysis of the relationship between repetitive arm movements and the development of specific shoulder diseases (Table 4). In the aforementioned cohort study [7], the doubling dose for an operatively-treated subacromial impingement syndrome when performing a variance-weighted linear regression of the exposure and odds ratio was reached at approximately 9404 h of highly-repetitive activity (37,616 h of moderately-repetitive activity).

#### 3.2.2. Sex-Specific Differences in Disease Risk

Dalbøge and colleagues [7] found comparable risk progressions for men and women in their cohort study; the sex-specific interaction term did not reach statistical significance. The corresponding nested case-control study (2017) [16] gave slightly higher risk estimates for men than for women, with comparable exposure levels in the highest exposure category. Overall, the Danish study did not provide any reliable evidence of sex-specific differences on the risk of illness with repetitive movements of the upper arm.

#### 3.2.3. Results of Other Studies

The cohort study by Bodin and colleagues (2012, no table) [10] did not find an increased risk of rotator cuff syndrome in neither men nor women during highly repetitive activities for at least four hours a day. However, neither the exact duration of the daily exposure nor the number of exposed shifts were taken into account. The cross-sectional study by Frost and colleagues (2002, no table) [2] found an association between repetitive shoulder movements with risk of shoulder tendonitis (OR = 3.3; 95% 1.3–8.1 at 15–36 movements per minute). Sex-specific risks were not specified, and the calculation of a cumulative exposure dose was not possible due to the cross-sectional design. This also applies to the cross-sectional study by Silverstein and coauthors (2008, no table) [13], which found no clear relationship between the frequency of shoulder movements and a rotator cuff syndrome when investigating men and women.

### 3.3. Forceful Shoulder Exertions

#### 3.3.1. Cumulative Exposure Calculation and Risk of Disease

The Danish cohort study [7,15] with its nested case-control study [16], and our own case-control study [3] allow for risk calculations for the cumulative load due to forceful shoulder exertions (Table 5). A variance-weighted regression analysis resulted in a doubling dose of approximately 55 “force-years” for the publication by Dalbøge and coauthors [7]. The doubling dose for the nested case-control study [16] was approximately 37 “force-years” for men and about 41 “force-years” for women. The categorized variance-weighted regression analysis in our own case-control study showed a doubling risk for lifting and carrying of a load weight of at least 20 kg with a cumulative duration of 217 h; in the continuous analysis using a cubic model (which had a considerably better model fit than a linear model as expressed by the AIC), the corresponding doubling dose was slightly lower at 194 h. The doubling dose values of the Danish study and the German study are based on considerably different measures, and thus a meta-analytical summary was not possible.

#### 3.3.2. Sex-Specific Differences in Disease Risk

Based on a graphic representation, Dalbøge and colleagues (2014, Figure 2) [7] found comparable risk progressions for men and women. In the highest exposure category, the risk estimate was slightly higher for women than for men, and the sex-specific interaction term reached statistical significance (*p* = 0.02) when the number of cases was very high. On the other hand, the nested case-control study [16] yielded slightly higher risk estimates for men than for women with comparable exposure levels in the individual exposure categories. Overall, the Danish study did not provide any reliable evidence of sex-specific differences in the dose-response curve for forceful shoulder exertions.

#### 3.3.3. Results of Other Studies

In their cohort study, Bodin and colleagues. (2012, no table) [10] found the rotator cuff syndrome to be more common in men than in women in activities that involve physical exertion (not precisely defined). In a separate univariate evaluation, the risk estimator achieved statistical significance in men, but not in women. The cross-sectional study by Miranda and colleagues (2005, no table) [12] showed partly statistically-significant increased risk estimates when moving loads weighing over 20 kg more than 10 times a day, but the risk progression did not increase monotonically. No risk estimates were given for men in this regard since load movements were not included in the final model. The cross-sectional study by Frost and coauthors (2002, no table) [2] found significantly increased disease risks in the joint analysis of men and women when they used at least 10% of the maximum strength (OR = 4.2; 95% CI 1.7–10.4). Also, the cross-sectional study by Silverstein and colleagues (2008, no table) [13] found a positive dose-response relationship between exertion of force and shoulder diseases when evaluating men and women together, a result that was not found in the cross-sectional study by Svendsen and colleagues [6].

In a recent cohort study (“Copenhagen Airport Study” [17,18]), male luggage carriers at Copenhagen Airport were compared with unskilled workers in the Copenhagen area. Subacromial shoulder diseases (ICD-10 M75.1–M75.5 and M75.8–M75.9) were determined by linking with the Danish patient registry. The length of employment as a luggage carrier was associated with the diagnosis of subacromial shoulder disease [18], although it was not statistically significant in the adjusted model. On the basis of the self-reported work history, abduction moments and force on the shoulders were estimated using biomechanical models. In the adjusted models [17] there were only increased risk estimates between 1.20 (for the highest category of the cumulative compression force and for the highest category of the cumulative abduction moment) and 1.25 (for the highest category of the cumulative supraspinatus force, 90th percentile). These risk estimates were not statistically significant.

### 3.4. Working with Vibration of the Hands and Arms

#### 3.4.1. Cumulative Exposure Calculation and Risk of Disease

In addition to the Danish cohort study [7,15] and its nested case-control study [16], the cohort study by Sutinen and colleagues [14] calculated a cumulative exposure dose (Table 6). With an extrapolation of the risk with regression analysis based on Dalbøge and colleagues [7], activities with moderate vibration acceleration (≥3–10 m/s^2^) resulted in a doubling dose of 5312 h (corresponding with a doubling dose of 10,624 h for activities with low vibration acceleration (<3 m/s^2^)). The study by Sutinen and colleagues [14] used considerably different exposure measurements, and therefore a meta-analytical summary was not possible.

#### 3.4.2. Sex-Specific Differences in Disease Risk

Dalbøge and colleagues (2014, Figure 2) [7] found comparable risk progressions for men and women, and the sex-specific interaction term did not reach statistical significance. The embedded case-control study by Dalbøge and colleagues [16] found a marginally-higher risk estimate for men than for women (1.9 vs. 1.8) with a somewhat higher cumulative exposure dose (approximately 11,500 vs. 8000 h of activity with low vibration acceleration). Overall, this study does not provide any indication of sex-specific differences in the risk of disease when exposed to hand-arm vibrations at work.

#### 3.4.3. Results of Other Studies

In their cohort study, Bodin and coauthors (2012, no table) [10] found no statistically significant increased risk of rotator cuff syndrome in men or women who had worked with vibrating hand-held tools for at least two hours per shift. Our own case-control study [3] showed a monotonic increase in the risk of a supraspinatus tendon rupture with increasing number of years in which hand-held vibrating tools were used. However, for this study, the daily usage time of the vibrating tools was unknown.

## 4. Discussion

The present systematic review with meta-analysis represents an update of the published work by van der Molen and colleagues [8], with the aim to determine the dose-response relationship and doubling dose values for physical workload (1. working with the hands at or above shoulder level, 2. working with repetitive movement of the upper arm at the shoulder joint, 3. forceful shoulder exertions, and 4. working with vibration of the hands and arms) and specific shoulder diseases. The pooled analysis of three studies [3,6,16] resulted in a 21% risk increase (95% CI 4–41%) per 1000 h of work above the shoulder. A “best-estimate” of the doubling dose is 3636 h of work with hands at or above shoulder level. For the other types of exposure, a meta-analytical summary was not possible either due to insufficient study numbers or different exposure measures. Based on the 2014 study by Dalbøge and colleagues [7], a doubling dose of approximately 9404 h can be estimated for highly repetitive activities of the upper arm at the shoulder joint. Based on the Danish cohort study, the doubling dose for activities requiring force in the shoulder area are 55 “force-years” [7], and 37 and 41 “force-years” for men and women, respectively [16] (1 “force-year” = working with a force intensity of 2 of 5 for one year). In our own case-control study [3] based on continuous exposure data, we calculated a 217 h doubling dose of cumulative duration of lifting and carrying operations (with a load weight of at least 20 kg). For work with hand-arm vibrations, the cohort study by Dalbøge and colleagues [7] resulted in an estimate of doubling dose of 5312 h of activity with moderate vibration acceleration (≥3–10 m/s^2^).

### 4.1. Strengths and Limitations

One of the strengths of the present work is that the update of the systematic review by van der Molen and colleagues [8] was carried out in accordance with published methodology; a double review (A.S. and U.B.-A.) of all new titles and abstracts, as well as full texts of the newly identified studies published up to November 2018 was also done. Quality assessment of the newly identified studies was done by two independent reviewers, as per the criteria used by van der Molen and colleagues [8]. A Danish cohort study [7] and its embedded case-control study [16] was identified for useful derivation of the dose-response relationship. This embedded case-control study distinguishes itself by the fact that the exposure data was estimated by measurements. Primary data was available for our own case control study [3], such that a dose-response relationship could be derived on the basis of continuous exposure data. For the other included studies, the dose-response relationship could only be estimated using published results with categorized exposure data.

### 4.2. Calculation of Cumulative Exposure

A limitation of the present systematic review is the heterogeneity of the exposure measures, which did not allow for a meta-analytical summary of studies for several exposures. In order to determine cumulative exposure, the mean exposure in the published exposure categories had to be estimated due to the lack of available primary data (with the exception of our own case-control study [3]). These estimates are, therefore, associated with uncertainties. Finally, for the Danish cohort study by Dalbøge and colleagues [7], the largest study so far, exposure data was only available for the last 10 years before diagnosis; the arbitrary assumption that entry into physically demanding work occurred at 20 years of age is associated with an information bias.

### 4.3. Heterogeneity of the Outcome Definitions

Another limitation of this systematic review lies in the heterogeneity of the outcome definitions, which were used to derive a dose-response relationship. While our own case-control study [3] as well as the cross-sectional study by Svendsen and colleagues [6] relied on rotator tendon lesions confirmed by MRI, the Danish cohort study and its nested case-control study [7,16] included a considerably broader spectrum of shoulder diseases treated with surgery (ICD-10 M75.1–M75.9: “shoulder lesions” without “adhesive capsulitis of the shoulder” and M19: “other and unspecified osteoarthritis”). However, the effect of this broad outcome definition on the risk estimators can be assessed as small: a sub-analysis of the Danish cohort study in cases with an operatively-treated rotator cuff lesion (ICD-10 M75.1 [23,24]) led to a slight increase in the risk estimates in the highest exposure categories for over-shoulder work (OR = 2.4; 95% 2.1–2.8 when limited to M75.1, compared to OR = 2.1; 95% CI 2.0–2.2 when all cases were included); for “repetition-years” (OR = 2.2; 95% CI 1.9–2.5 when limited to M75.1, compared to OR = 1.9; 95% CI 1.8–2.0 when all cases were included); for “force-years” (OR = 1.9; 95% CI 1.6–2.2 when limited to M75.1, compared to OR = 1.7; 95% CI 1.6–1.8 when all cases were included); and for “hand-arm vibration years” (OR = 1.6; 95% CI 1.4–1.8 when limited to M75.1, compared to OR = 1.5; 95% CI 1.5–1.6 when all cases were included). It can be thus assumed that a restriction to rotator cuff lesions will result in a slight reduction in the respective doubling dose for all considered types of workload exposures.

### 4.4. Assessment of Study Quality

Only studies with a quality score of at least 13 out of 16 were included in the assessment of the dose-response relationship or the doubling dose. All studies with a (arbitrarily-chosen) score of ≥11 out of 16 were regarded as “low risk of bias” in the previous review by van der Molen and colleagues [8]. Even though, in our post-evaluation, we partly arrived at slightly differing results in the studies previously evaluated by the van der Molen review, we kept their original assessment and supplemented a quality assessment of the newer studies only. In the recently published systematic review by Dalbøge and colleagues [25], which included studies published up to October 2016, the same criteria were used. This review also found slightly different quality ratings than in the review by van der Molen [8], which did not lead to changes in the overall assessment of the studies (low or high risk of bias). In general, the methods used by van der Molen and colleagues [8] to generate the total score can be criticized because a study with a serious methodological error (for example, a study lacking a defined study base or source population) could still receive a “15 out of 16” score and thus incorrectly be rated as a “low risk of bias” study. However, no such serious methodological errors could be identified in the studies that we included in the derivation of the dose-response relationship.

### 4.5. Biological Plausibility

The finding of a positive dose-response relationship between cumulative physical loads on the shoulder joint and the development of a specific shoulder disease—in particular a rotator cuff lesion —is biologically plausible against the background of human and animal experiments. Hagberg [26] was able to show that muscle abduction or anteversion in the shoulder joint by 90° results in muscle fatigue in the supraspinatus muscle after approximately 5 min. In welders, this fatigue was confirmed in the supraspinatus muscle when the hand was held above the shoulder or at head level [27,28]. An abduction in the shoulder joint of 30° results in a significant increase in pressure in the area of the supraspinatus muscle to 5.6 kPa (42 mmHg). Such pressure leads to a significant decrease in intramuscular blood flow in the supraspinatus muscle [29,30]. Lifting a load of one and two kilograms through an abduction of the shoulder joint leads to a significantly greater decrease in intramuscular blood flow in the supraspinatus muscle than through an abduction without a weight load [31,32,33]. Furthermore, a pathologic-anatomical study showed that the tendon of the supraspinatus muscle comes under pressure at a 60° anteversion between the humerus and the acromion and the coracoacromial ligament. With simultaneous internal rotation, this is already the case with an anteversion of 30°. Furthermore the study showed that the tendon of the biceps brachii muscle already comes under pressure with an anteversion of 15° between the humerus and the coracoacromial ligament [34]. In addition, various animal studies have indicated that the repetitive movement of the supraspinatus tendon leads to inflammatory changes and signs of wear that reduce the resilience of the tendon [35,36,37]. These results were confirmed by an experimental investigation which, after repetitive loading, also demonstrated inflammatory changes and signs of wear in other tendons [38,39,40,41,42,43].

### 4.6. Comparison of the Dose-Response Association in Men and Women

For over-shoulder work, repetitive movements, and hand-arm vibrations, the Danish cohort study [7] found no significant differences in risk progression for men and women. For forceful shoulder exertions, this cohort [7] showed slightly higher risk estimates for women than for men, while conversely, the nested case-control study showed slightly higher risk estimates for men than for women [16]. Overall, based on the large Danish cohort study in men and women, comparable dose-response progressions can be assumed for the association between physical workload and specific shoulder diseases. The systematic review by Dalbøge and colleagues [25] also found no clear evidence of sex-specific disease risks: although over-shoulder work and exertion in the shoulder area tended to have slightly higher risk estimates for women than for men, this finding was not supported by high-quality studies. There is a preventive importance of reducing occupational physical demands, equally important for men and women, not only to avoid the onset of disease but also for the prevention of early retirement due to illness. This was clearly demonstrated by a recently-published Finnish study by Sirén and colleagues [44]: 46% and 41% of early retirement due to shoulder disease in men and women, respectively, is attributed to work-related physical demands.

### 4.7. Course of the Dose-Response Association between the Cumulative Load and Specific Shoulder Diseases

A meta-analytical investigation of non-linear risk progression was not possible due to the small number of studies which investigated the risk progression between cumulative physical workload and specific shoulder diseases. It was, therefore, not possible to examine the question of any “threshold values” for exposure below which there is no increased risk of specific shoulder diseases. In a recently published analysis of the Danish cohort study [15], the risk of shoulder disease depending on the exposure time to loads of different intensities was investigated. The authors found evidence that below a median upper arm velocity of 45°/s, there is no increased risk of repetitive activities. However, the exertion of force ≥10% of the maximum arbitrary force as well as an upper arm elevation of >90° for more than 2 min per day were already associated with an increased risk of shoulder disease.

For the meta-analysis on work with hands above the shoulder level, we used a linear regression model for available continuous data from our own case-control study [3]. A quadratic or cubic model for the continuous data resulted only in a slightly better fit, with an almost unchanged value of doubling dose. For forceful work using the shoulders, the doubling dose using our own case-control data was estimated using a cubic model, as, in this case, its fit was clearly superior to a linear or quadratic model.

## 5. Conclusions

This systematic review—an update of the van der Molen et al. (2017) review—was able to derive the dose-response relationship, and thus the doubling dose for work at or above shoulder level and specific shoulder diseases, by pooling three studies. For repetitive movements, the use of force in the shoulder area, and hand-arm vibrations, the dose-effect relationship could only be estimated on the basis of individual studies. A meta-analytical summary of the risk estimators was not possible due to insufficient study numbers or different exposure measures. Based only on few studies, no evidence for sex-specific differences in the dose-response relationship was found. Overall, the present systematic review with meta-analysis can contribute to the question regarding the level of exposure above which specific shoulder diseases—in particular rotator cuff lesions—should be recognized as an occupational disease.

## Figures and Tables

**Table 1:** Definition and assessment of exposure and outcomes for studies included (only publications not mentioned by van der Molen et al. [8]).

Author, Study Design ^1^	Outcome (Prevalence; Incidence)		Exposure	
	Definition	Assessment	Definition	Assessment
Dalbøge et al. 2018 [15] cohort; Svendsen 2017 [23]	As in Dalbøge et al. 2014 [7]: Subacromial impingement syndrome first-time surgery 2003–08 ICD-10: M19 or M75.1–M75.9); Svendsen 2017 [23]: only rotator cuff tear or rupture (M75.1)	Medical registry	Job exposure matrix assessments (10-yr time window with a 1-yr lag time) of years of arm elevation (>90°), repetitiveness, force and acceleration (HAV), shoulder load by five specialists in occupational medicine	Registries (years till 1993), expert assessment, job exposure matrix
Dalbøge et al. 2017 [16]. nested CC (2 age- and sex matched controls per case), in cohort described by Dalbøge et al. 2014 [7]	As in Dalbøge et al. 2014 [7]: Subacromial impingement syndrome first-time surgery 2003-08 ICD-10: M19 or M75.1–M75.9)	Medical registry	Updated job exposure matrix (JEM) assessments (10-yr time window with a 1-yr lag time) of years of arm elevation (>90°), repetitiveness, force and acceleration (HAV), shoulder load by five specialists in occupational medicine; job exposure matrix assessments up to the index year (yr. of the surgery of the case)	Measurement-based JEM (related to self-reported job titles, up to 6 in a 10-year period)
Møller et al. 2018 [17]. Cohort study (Copenhagen Airport Cohort, see Thygesen et al. 2016 [18])	Diagnosis or surgical treatment of subacromial shoulder disorders (ICD 75.1–75.5, 75.8, 75.9)	National patient register	Accumulated abduction moment, compression force or supraspinatus force and seniority as baggage handler	Capture motion system, ground reaction force, biomechanical modelling and company records
Thygesen et al. [18].Cohort study (Copenhagen Airport Cohort) based on unskilled men at Copenhagen Airport & in the Greater Copenhagen area	Diagnosis or surgical treatment of subacromial shoulder disorders (ICD 75.1–75.5, 75.8, 75.9)	National patient register	Seniority as baggage handler	Company and union records

^1^ CC = case control study, CS = cross-sectional study.

**Table 2:** Methodological quality scores of 16 items for studies regarding risk factors (only publications not mentioned by van der Molen et al. 2017 [8]).

Authors/Quality Item	1 (Study Groups De-fined)	2 (par-tici-pa-tion ≥70%)	3 (Num-ber Case ≥50)	4 (Expo-sure Measure-ment)	5 (Dose-Re-sponse)	6 (Blind for Out-come Sta-tus)	7 (Out-come Defini-tion)	8 (Assess-ment Method)	9 (Blind for Expo-sure Status)	10 (longitu-dinal Study Design)	11 (Inclu-sion and Exclu-sion Criteria)	12 (Fol-low-up Period ≥1 year)	13 (Info complet-ers Versus With-drawal)	14 (Data Presentation)	15 (Considera-tion of Confounders)	16 (Control for Confound-ing)	Sum
Thygesen et al. 2016 [18]	+	+	+	+	+	+	+	+	+	+	+	+	+	+	+	+ §	16
Møller et al. 2018 [17]	+	+	+	+	+	+	+	+	+	+	+	+	+	+	+	+ §	16
Dalbøge et al. 2018 [15]	+	+	+	+	+	+	+	+	+	+	+	+	+	+	+	+ §§	16
Dalbøge et al. 2017 [16]	+	−	+	+	+	+	+	+	+	+	+	+	− *	+	+	+ §§§	14

§ Adjusted for age, sex, calendar year, educational level, pre-employment shoulder injury, use of lifting equipment and use of extendable belt loaders in the aircraft baggage compartments; §§ adjusted for age, sex, region, calendar year at start follow-up, number of the particular follow-up year, durations of exposure in the two other intensity categories above minimal, fully adjusted models: additionally cumulative exposures to other occupational mechanical exposures. * just stated: “minor differences with respect to age and sex distribution” (p. 731). §§§ matched for age and sex; adjusted for (present) body mass index, pack-years of smoking, leisure time shoulder intensive sports, diabetes mellitus, psychosocial strain, social support, region of residence.

**Table 3:** Working with the hands at or above shoulder level.

Study; Outcome	Exposure Parameter	Calculation of Lifetime Hours	Cumulative Exposure (Lifetime Hours)	OR Adjusted for Non-Occupational Factors (95% CI)
Dalbøge et al. 2014 [7] *	**Arm-Elevation-Years** (1 Arm-Elevation-Year = Working with Elevated Arm(s) >90° for 0.5 h/day for 1 Year)			
**Outcome**: subacromial impingement syndrome first-time surgery 2003-08 ICD-10: M19 or M75.1–M75.9. **Men and women** (*n* = 2,374,403 participants, of those 14,118 cases)	0	0 h	0 h	1.0 -
	>0–2	3 × 1 × 0.5 h/day × 220 days	330 h	1.4 (1.4–1.5)
	>2–5	3 × 3.5 × 0.5 h/day × 220 days	1155 h	1.5 (1.5–1.6)
	>5–10	3 × 7.5 × 0.5 h/day × 220 days	2475 h	1.8 (1.7–1.9)
	>10–56	3 × 15 × 0.5 h/day × 220 days	4950 h	2.1 (2.0–2.2)
**Svendsen 2017 [23] (Personal Communication; Same Results Given in Dalbøge et al. 2019a [24])**				
**Outcome:** rotator cuff lesion ICD10: M75.1. **Men and women**	0	0 h	0 h	1.0 -
	>0–2	3 × 1 × 0.5 h/day × 220 days	330 h	1.5 (1.3–1.7)
	>2–5	3 × 3.5 × 0.5 h/day × 220 days	1155 h	1.6 (1.4–1.9)
	>5–10	3 × 7.5 × 0.5 h/day × 220 days	2475 h	1.9 (1.7–2.2)
	>10–56	3 × 15 × 0.5 h/day × 220 days	4950 h	2.4 (2.1–2.8)
Dalbøge et al. 2017 [16] **	**Arm-Elevation-Years** (Calibrated into ‘Predicted Measured Job Exposures’, 1 Arm-Elevation-Year Adds 4.8 min to the Background Duration of 2.3 min per Day with the Arm Elevated >90°)			
**Outcome:** subacromial impingement syndrome first-time surgery 2003-08 ICD-10: M19 or M75.1–M75.9. **Men** (*n* = 701 cases, 974 control subjects)	0	>0 h (background duration)	0 h	1.0 -
	>0–10 (mean 3.7)	3.6 × (3.7 × 7.1 min./day × 220 days)/60 min./h	347 h	2.0 (1.6–2.5)
	>10–60 (mean 23.5)	3.6 × (23.5 × 7.1 min./day × 220 days)/60 min./h	2202 h	2.3 (1.8–3.0)
**Women** (*n* = 863 cases, 1260 control subjects)	0	>0 h (background duration	0 h	1.0 -
	>0–10 (mean 4.1)	3.4 × (4.1 × 7.1 min./day × 220 days)/60 min./h	363 h	1.6 (1.3–1.9)
	>10–60 (mean 22.2)	3.4 × (22.2 × 7.1 min./day × 220 days)/60 min./h	1965 h	1.9 (1.4–2.6)
**Seidler et al. 2011a [3]**	**Cumulative Work Above Shoulder Level [Hrs.]**			
**Outcome**: supraspinatus lesion. **Men***(n = 483 cases, n = 300 control subjects)*	No work above shoulder level		0 h	1.0
	>0–<610 h (median 272 h)$		272 h	1.7 (1.0–2.8)
	610–<3195 h (median 1529 h)$		1529 h	2.6 (1.6–4.2)
	3195–64,057 h (median 9965 h) $		9965 h	4.1 (2.6–6.4)
	Per 1000 h. work above shoulder level (based on continuous variable)			1.09 (1.05–1.13) $
**Svendsen et al. 2004b [6]**	**Cumulative Duration (Month) of Upper Arm Elevation > 90°**			
**Outcome:** supraspinatus tendonitis. **Men** (*n* = 52 cases)	<10	5 × 20 days × 8 h	800 h	1.00
	10–<20	15 × 20 days × 8 h	2400 h	0.95 (0.41–2.20)
	≥20	30 × 20 days × 8 h	4800 h	2.33 (0.93–5.84)

* Dalbøge et al. 2014 [7] adjusted for time varying age (five categories), sex, region of residence (five regions), calendar year at start of follow up. ** Dalbøge et al. 2017 [16] individually matched on sex, date of birth, adjusted for BMI, smoking (PY), leisure time shoulder intensive sports, diabetes mellitus, psychosocial strain, region of residence and additionally adjusted for demands and control. $ own calculation; OR for continuous variable adjusted for age, region, apparatus gymnastics/shot put/javelin/hammer throwing/wrestling and tennis. ‡ MRI: magnetic resonance imaging.

**Table 4:** Working with repetitive movement of the upper arm at the shoulder joint.

Study; Outcome	Exposure Parameter	Calculation of Lifetime Hours	Cumulative Exposure (Lifetime Hours of Highly Repetitive Work) †	OR Adjusted for Non-Occupational Factors (95% CI)
Dalbøge et al. 2014 [7] *	**Repetition Years** (1 Repetition Year = Performing Moderately Repetitive Work for 4 h/day for 1 year or Highly Repetitive Work for 1 h/day for 1 Year)			
**Outcome:** subacromial impingement syndrome first-time surgery 2003-08 ICD-10: M19 orM75.1–M75.9. **Men and women** (*n* = 2,374,403 participants, of those 14,118 cases)	0	0 h	0 h	1.0
	>0–1	3 × 0.5 × 1 h/day × 220 days	330 h	1.2 (1.1–1.3)
	>1–2	3 × 1.5 × 1 h/day × 220 days	990 h	1.5 (1.5–1.6)
	>2–10	3 × 6 × 1 h/day × 220 days	3960 h	1.6 (1.5–1.6)
	>10–68	3 × 18 × 1 h/day × 220 days	11,880 h	1.9 (1.8–2.0)
Dalbøge et al. 2017 [16] **.*N* = 1564 Cases, 2234 Control Subjects	**Repetition–Years** (Calibrated into ‘Predicted Measured Job Exposures’, 1 Repetition Year Adds 25°/s to the Background Median Angular Velocity of Upper Arm Movements of 27°/s per day)			
**Outcome:** subacromial impingement syndrome first-time surgery 2003-08 ICD-10: M19 or M75.1–M75.9. **Men** (*n* = 701 cases, 974 control subjects)	0			1.0 -
	>0–10 (mean 4.8)			1.7 (1.4–2.1)
	>10–17.5 (mean 12.7)			2.6 (1.5–4.6)
**Women** (*n* = 863 cases, 1260 control subjects)	0			1.0 -
	>0–10 (mean 5.5)			1.5 (1.2–1.9)
	>10–17.5 (mean 11.8)			2.0 (1.0–4.4)

* Dalbøge et al. (2014) [7] adjusted for time varying age (five categories), sex, region of residence (five regions), calendar year at start of follow up. † hours of highly repetitive work are given for Dalbøge et al. (2014). ** Dalbøge et al. (2017) [16] individually matched on sex, date of birth, adjusted for BMI, smoking (PY), leisure time shoulder intensive sports, diabetes mellitus, psychosocial strain, region of residence and additionally adjusted for demands and control.

**Table 5:** Forceful shoulder exertions.

Study; Outcome	Exposure Parameter	Calculation of Lifetime Duration	Cumulative Exposure (Lifetime Hours Resp. Years)	OR Adjusted for Non-Occupational Factors (95% CI)
Dalbøge et al. 2014 [7] *	**Force-years** (1 Force-Year = Working with a Force Score of 2 (Five-Point Rating of Intensity Of Exertion, Moore, J.S.; Garg, A. The Strain Index: a Proposed Method to Analyze Jobs for Risk Of Distal Upper Extremity Disorders. *Am. Ind. Hyg. Assoc. J.* 1995, *56*:, 443–58) for 1 year)			
**Outcome:** subacromial impingement syndrome first-time surgery 2003-08 ICD-10: M19 or M75.1–M75.9. **Men and women** (*n* = 2,374,403 participants, of those 14,118 cases)	<5	0 yrs.	0 yrs.	1.0 -
	5	3 × 2.5 yrs.	7.5 yrs.	0.7 (0.6–0.7)
	>5–7.5	3 × 6.25 yrs.	18.8 yrs.	1.2 (1.1–1.2)
	>7.5–10	3 × 8.75 yrs.	26.3 yrs.	1.5 (1.4–1.6)
	>10–20	3 × 12.5 yrs.	37.5 yrs.	1.7 (1.6–1.8)
Dalbøge et al. 2017 [16] **	**Force-Years** (1 Force-Year = Working with a Force Score of 2 (Five-Point Rating of Intensity of Exertion, Moore, J.S.; Garg, A. The strain index: a proposed method to analyze jobs for risk of distal upper extremity disorders. *Am. Ind. Hyg. Assoc. J.* 1995, *56*, 443–458) for 1 Year)			
**Outcome:** subacromial impingement syndrome first-time surgery 2003-08 ICD-10: M19 or M75.1–M75.9. **Men** (*n* = 701 cases, 974 control subjects)	0	0 yrs.	0 yrs.	1.0 -
	>0–10 (mean 5.7)	3.6 × 5.7 yrs.	20.5 yrs.	2.0 (1.6–2.5)
	>10–30 (mean 16.6)	3.6 × 16.6 yrs.	59.8 yrs.	2.6 (2.0–3.4)
**Women** (*n* = 863 cases, 1260 control subjects)	0	0 yrs.	0 yrs.	1.0 -
	>0–10 (mean 6.4)	3.4 × 6.4 yrs.	21.8 yrs.	1.7 (1.4–2.1)
	>10–30 (mean 17.7)	3.4 × 17.7 yrs.	60.2 yrs.	2.3 (1.6–3.3)
**Seidler et al. 2011a [3]**	**Cumulative Lifting and Carrying of Loads ≥ 20 kg [hours]**			
**Outcome**: supraspinatus lesion. **Men** (*n* = 483 cases, *n* = 300 control subjects)	No lifting and carrying of loads ≥ 20 kg [h]		0 h	1.0
	>0–<9.6 h (median 3.2 h) $		3.2 h	1.4 (0.8–2.4)
	9.6–<77 h (median 28 h) $		28 h	2.0 (1.2–3.3)
	77–9038 h (median 385 h) $		385 h	3.3 (2.1–5.2)

* Dalbøge et al. (2014) [7] adjusted for time varying age (five categories), sex, region of residence (five regions), calendar year at start of follow up. ** Dalbøge et al. (2017) [16] individually matched on sex, date of birth, adjusted for BMI, smoking (PY), leisure time shoulder intensive sports, diabetes mellitus, psychosocial strain, region of residence and additionally adjusted for demands and control. $ own calculation.

**Table 6:** Working with vibration of the hands and arms.

Study; Outcome	Exposure Parameter	Calculation of Lifetime Hours	Cumulative Exposure (Lifetime Hours of Moderate Acceleration)	OR Adjusted for Non-Occupational Factors (95% CI)
Dalbøge et al. 2014 [7] *	**HAV-Years** (1 HAV-Year = Working with a Hand-Held Vibrating Tool with Low Acceleration for 1 h/day for 1 year, or Working with a Hand-Held Vibrating Tool with Moderate Acceleration for 0.5 h/day for 1 year; Low, Moderate and High Acceleration was Defined as <3, ≥3–10 and >10 m/s^2^, Duration was Rated in Half-Hour Intervals)			
**Outcome:** subacromial impingement syndrome first-time surgery 2003-08 ICD-10: ML19 or M75.1–M75.9. **Men and women** (*n* = 2,374,403 participants, of those 14,118 cases)	0	0 h	0 h	1.0 -
	<0–5	3 × 2.5 × 0.5 h/day × 220 days	825 h	1.3 (1.2–1.3)
	>5–58	3 × 10 × 0.5 h/day × 220 days	3300 h	1.5 (1.5–1.6)
Dalbøge et al. 2017 [16] **	**HAV-Years** (1 HAV-Year = Working with a Hand-Held Vibrating Tool with Low Acceleration for 1 h/day for 1 year, or Working with a Hand-Held Vibrating Tool with Moderate Acceleration for 0.5 h/day for 1 year; Low, Moderate and High Acceleration was Defined As <3, ≥3–10 and >10 m/s^2^, Duration was Rated in Half-Hour Intervals)			
**Outcome:** subacromial impingement syndrome first-time surgery 2003-08 ICD-10: M19 or M75.1–M75.9. **Men** (*n* = 701 cases, 974 control subjects)	0	0 h	0 h	1.0 -
	>0–58 (mean 14.5)	3.6 × 14.5 × 0.5 h/day × 220 days	5742 h	1.9 (1.5–2.4)
**Women** (*n* = 863 cases, 1260 control subjects)	0	0 h	0 h	1.0 -
	>0–20 (mean 10.6)	3.4 × 10.6 × 0.5 h/day × 220 days	3964 h	1.8 (1.3–2.6)
**Sutinen et al. 2006 [14]**	**Lifelong vibration energy** [modified from Bovenzi M, Franzinelli A, Mancini R, Cannava MG, Maiorano M, Ceccarelli F (1995) Dose–response relation for vascular disorders induced by vibration in the fingers of forestry workers. Occup Environ Med 52:722–730]			
**Outcome:** rotator cuff syndrome. **Men** (*n* = 52 participants at 11 surveys, of those *n* = 10 cases)	Per 1 unit of lifelong vibration energy?			1.04 (1.00–1.07) *p* = 0.032

* Dalbøge et al. (2014) [7] adjusted for time varying age (five categories), sex, region of residence (five regions), calendar year at start of follow up. ** Dalbøge et al. (2017) [16] individually matched on sex, date of birth, adjusted for BMI, smoking (PY), leisure time shoulder intensive sports, diabetes mellitus, psychosocial strain, region of residence and additionally adjusted for demands and control.

## Supplementary Materials

The following are available online at https://www.mdpi.com/1660-4601/17/4/1243/s1, Table S1: Reasons for exclusion of studies. Table S2: Above-shoulder work sensitivity analyses. Figure S1: Forest plot of shoulder disease risk due to work above shoulder level (sensitivity analysis 1). Figure S2: Forest plot of shoulder disease risk due to work above shoulder level (sensitivity analysis 2). Figure S3: Forest plot of shoulder disease risk due to work above shoulder level (sensitivity analysis 3).

## References

[1] Frost P., Andersen J.H. (1999). Shoulder impingement syndrome in relation to shoulder intensive work. Occup. Environ. Med..

[2] Frost P., Bonde J.P.E., Mikkelsen S., Andersen J.H., Fallentin N., Kaergaard A., Thomsen J.F. (2002). Risk of shoulder tendinitis in relation to shoulder loads in monotonous repetitive work. Am. J. Ind. Med..

[3] Seidler A., Bolm-Audorff U., Petereit-Haack G., Ball E., Klupp M., Krauss N., Elsner G. (2011). Work-related lesions of the supraspinatus tendon: A case–control study. Int. Arch. Occup. Environ. Health.

[4] Svendsen S.W., Bonde J.P., Mathiassen S.E., Stengaard-Pedersen K., Frich L. (2004). Work related shoulder disorders: Quantitative exposure-response relations with reference to arm posture. Occup. Environ. Med..

[5] Svendsen S.W., Dalbøge A., Andersen J.H., Thomsen J.F., Frost P. (2013). Risk of surgery for subacromial impingement syndrome in relation to neck-shoulder complaints and occupational biomechanical exposures: A longitudinal study. Scand. J. Work Environ. Health.

[6] Svendsen S.W., Gelineck J., Mathiassen S.E., Bonde J.P., Frich L.H., Stengaard-Pedersen K., Egund N. (2004). Work above shoulder level and degenerative alterations of the rotator cuff tendons: A magnetic resonance imaging study. Arthritis Rheum. Off. J. Am. Coll. Rheumatol..

[7] Dalbøge A., Frost P., Andersen J.H., Svendsen S.W. (2014). Cumulative occupational shoulder exposures and surgery for subacromial impingement syndrome: A nationwide danish cohort study. Occup. Environ. Med..

[8] Van der Molen H.F., Foresti C., Daams J.G., Frings-Dresen M.H., Kuijer P.P.F. (2017). Work-related risk factors for specific shoulder disorders: A systematic review and meta-analysis. Occup. Environ. Med..

[9] Seidler A., Euler U., Bolm-Audorff U., Ellegast R., Grifka J., Haerting J., Jäger M., Michaelis M., Kuss O. (2011). Physical workload and accelerated occurrence of lumbar spine diseases: Risk and rate advancement periods in a german multicenter case-control study. Scand. J. Work Environ. Health.

[10] Bodin J., Ha C., Le Manac’h A.P., Sérazin C., Descatha A., Leclerc A., Goldberg M., Roquelaure Y. (2012). Risk factors for incidence of rotator cuff syndrome in a large working population. Scand. J. Work Environ. Health.

[11] Herin F., Vézina M., Thaon I., Soulat J.-M., Paris C. (2012). Predictors of chronic shoulder pain after 5 years in a working population. PAIN^®^.

[12] Miranda H., Viikari-Juntura E., Heistaro S., Heliövaara M., Riihimäki H. (2005). A population study on differences in the determinants of a specific shoulder disorder versus nonspecific shoulder pain without clinical findings. Am. J. Epidemiol..

[13] Silverstein B.A., Bao S.S., Fan Z.J., Howard N., Smith C., Spielholz P., Bonauto D., Viikari-Juntura E. (2008). Rotator cuff syndrome: Personal, work-related psychosocial and physical load factors. J. Occup. Environ. Med..

[14] Sutinen P., Toppila E., Starck J., Brammer A., Zou J., Pyykkö I. (2006). Hand-arm vibration syndrome with use of anti-vibration chain saws: 19-year follow-up study of forestry workers. Int. Arch. Occup. Environ. Health.

[15] Dalbøge A., Frost P., Andersen J.H., Svendsen S.W. (2018). Surgery for subacromial impingement syndrome in relation to intensities of occupational mechanical exposures across 10-year exposure time windows. Occup. Environ. Med..

[16] Dalbøge A., Frost P., Andersen J.H., Svendsen S.W. (2017). Surgery for subacromial impingement syndrome in relation to occupational exposures, lifestyle factors and diabetes mellitus: A nationwide nested case–control study. Occup. Environ. Med..

[17] Møller S.P., Brauer C., Mikkelsen S., Alkjær T., Koblauch H., Pedersen E.B., Simonsen E.B., Thygesen L.C. (2018). Risk of subacromial shoulder disorder in airport baggage handlers: Combining duration and intensity of musculoskeletal shoulder loads. Ergonomics.

[18] Thygesen L.C., Mikkelsen S., Pedersen E.B., Møller K.L., Alkjær T., Koblauch H., Simonsen E.B., Møller S.P., Brauer C. (2016). Subacromial shoulder disorders among baggage handlers: An observational cohort study. Int. Arch. Occup. Environ. Health.

[19] Ijaz S., Verbeek J., Seidler A., Lindbohm M.-L., Ojajärvi A., Orsini N., Costa G., Neuvonen K. (2013). Night-shift work and breast cancer—A systematic review and meta-analysis. Scand. J. Work Environ. Health.

[20] Orsini N., Bellocco R., Greenland S. (2006). Generalized least squares for trend estimation of summarized dose–response data. Stata J..

[21] Aune D., Sen A., Prasad M., Norat T., Janszky I., Tonstad S., Romundstad P., Vatten L.J. (2016). Bmi and all cause mortality: Systematic review and non-linear dose-response meta-analysis of 230 cohort studies with 3.74 million deaths among 30.3 million participants. BMJ.

[22] Guyatt G., Oxman A.D., Akl E.A., Kunz R., Vist G., Brozek J., Norris S., Falck-Ytter Y., Glasziou P., deBeer H. (2011). Grade guidelines: 1. Introduction—Grade evidence profiles and summary of findings tables. J. Clin. Epidemiol..

[23] Svendsen S.W. (2017). Personal communication.

[24] Dalbøge A., Frost P., Andersen J.H., Svendsen S.W. (2019). Exposure–response relationships between cumulative occupational shoulder exposures and different diagnoses related to surgery for subacromial impingement syndrome. Int. Arch. Occup. Environ. Health.

[25] Dalbøge A., Svendsen S.W., Frost P., Andersen J.H. (2019). Association between Occupational Mechanical Exposures and Subacromial Impingement Syndrome: A Reference Document. https://www.google.com/url?sa=t&rct=j&q=&esrc=s&source=web&cd=7&ved=2ahUKEwjRp6W0iM_nAhWNFMAKHSCsDL4QFjAGegQIBxAB&url=https%3A%2F%2Fwww.aes.dk%2F-%2Fmedia%2F3EA35B61FFE3472EA738F8F4B909BCA7.ashx&usg=AOvVaw1w-un--_wZMqK7aHpHJMeF.

[26] Hagberg M. (1981). Electromyographic signs of shoulder muscular fatigue in two elevated arm positions. Am. J. Phys. Med..

[27] Herberts P., Kadefors R. (1976). A study of painful shoulder in welders. Acta Orthop. Scand..

[28] Kadefors R., Petersen I., Herberts P. (1976). Muscular reaction to welding work: An electromyographic investigation. Ergonomics.

[29] Järvholm U., Palmerud G., Styf J., Herberts P., Kadefors R. (1988). Intramuscular pressure in the supraspinatus muscle. J. Orthop. Res..

[30] Järvholm U., Styf J., Suurkula M., Herberts P. (1988). Intramuscular pressure and muscle blood flow in supraspinatus. Eur. J. Appl. Physiol. Occup. Physiol..

[31] Järvholm U., Palmerud G., Herberts P., Högfors C., Kadefors R. (1989). Intramuscular pressure and electromyography in the supraspinatus muscle at shoulder abduction. Clin. Orthop. Relat. Res..

[32] Järvholm U., Palmerud G., Karlsson D., Herberts P., Kadefors R. (1991). Intramuscular pressure and electromyography in four shoulder muscles. J. Orthop. Res..

[33] Palmerud G., Forsman M., Sporrong H., Herberts P., Kadefors R. (2000). Intramuscular pressure of the infra-and supraspinatus muscles in relation to hand load and arm posture. Eur. J. Appl. Physiol..

[34] Burns W.C., Whipple T. (1993). Anatomic relationships in the shoulder impingement syndrome. Clin. Orthop. Relat. Res..

[35] Perry S.M., McIlhenny S.E., Hoffman M.C., Soslowsky L.J. (2005). Inflammatory and angiogenic mrna levels are altered in a supraspinatus tendon overuse animal model. J. Shoulder Elb. Surg..

[36] Soslowsky L., Thomopoulos S., Tun S., Flanagan C., Keefer C., Mastaw J., Carpenter J. (2000). Neer award 1999: Overuse activity injures the supraspinatus tendon in an animal model: A histologic and biomechanical study. J. Shoulder Elb. Surg..

[37] Soslowsky L.J., Thomopoulos S., Esmail A., Flanagan C.L., Iannotti J.P., Williamson J.D., Carpenter J.E. (2002). Rotator cuff tendinosis in an animal model: Role of extrinsic and overuse factors. Ann. Biomed. Eng..

[38] Backman C., Boquist L., Fridén J., Lorentzon R., Toolanen G. (1990). Chronic achilles paratenonitis with tendinosis: An experimental model in the rabbit. J. Orthop. Res..

[39] Barbe M.F., Barr A.E., Gorzelany I., Amin M., Gaughan J.P., Safadi F.F. (2003). Chronic repetitive reaching and grasping results in decreased motor performance and widespread tissue responses in a rat model of msd. J. Orthop. Res..

[40] Barbe M.F., Elliott M.B., Abdelmagid S.M., Amin M., Popoff S.N., Safadi F.F., Barr A.E. (2008). Serum and tissue cytokines and chemokines increase with repetitive upper extremity tasks. J. Orthop. Res..

[41] Nakama L.H., King K.B., Abrahamsson S., Rempel D.M. (2007). Effect of repetition rate on the formation of microtears in tendon in an in vivo cyclical loading model. J. Orthop. Res..

[42] Nakama L.H., King K.B., Abrahamsson S., Rempel D.M. (2005). Evidence of tendon microtears due to cyclical loading in an in vivo tendinopathy model. J. Orthop. Res..

[43] Fedorczyk J.M., Barr A.E., Rani S., Gao H.G., Amin M., Amin S., Litvin J., Barbe M.F. (2010). Exposure-dependent increases in il-1β, substance p, ctgf, and tendinosis in flexor digitorum tendons with upper extremity repetitive strain injury. J. Orthop. Res..

[44] Sirén M., Viikari-Juntura E., Arokoski J., Solovieva S. (2019). Physical and psychosocial work exposures as risk factors for disability retirement due to a shoulder lesion. Occup. Environ. Med..

