# Supplementary material for: Dose–Response Relationship between Physical Workload and Specific Shoulder Diseases—A Systematic Review with Meta-Analysis
Source: Int J Environ Res Public Health. 2020 Feb 14;17(4):1243. doi: 10.3390/ijerph17041243 (PMC7068556; doi:10.3390/ijerph17041243)

Supplementary Table S1. Reasons for exclusion of studies.

Study authors	Reasons for exclusion
Alvarez-Rincon et al. 2018 [S1]	Only abstract available
Bovenzi et al. 2016 [S2]	No information on outcomes rotator cuff syndrome/subacromial impingment syndrome given
Castillo-Lozano 2017 [S3]	No information on outcomes rotator cuff syndrome/subacromial impingment syndrome given
Dalbøge et al. 2016 [S4]	No information on outcomes given
Haugsboe et al. 2018 [S5]	No information on outcomes rotator cuff syndrome/subacromial impingment syndrome given
Keener et al. 2017 [S6]	No information on included exposures, e.g. hand above shoulder level
Klussmann et al. 2017 [S7]	No information on outcomes rotator cuff syndrome/subacromial impingment syndrome given
Lubiatowski et al. 2018 [S8]	Missing diagnosis of rotator cuff syndrom in control subjects
Saleem et al. 2018 [S9]	No information on outcomes rotator cuff syndrome/subacromial impingment syndrome given
Thorlund Jakobsen et al. 2018 [S10]	No information on outcomes rotator cuff syndrome/subacromial impingment syndrome given

References

S1. Alvarez-Rincón, D.; Perez, N. 1054 physical workload exposure threshold in cumulative-trauma disorders useful for primary prevention and for causal assessment: A 12.5 y follow-up cohort study. BMJ Publishing Group Ltd: 2018.

S2. Bovenzi, M.; Prodi, A.; Mauro, M. A longitudinal study of neck and upper limb musculoskeletal disorders and alternative measures of vibration exposure. *International archives of occupational and environmental health* **2016**, *89*, 923-933.

S3. Castillo-Lozano, R. Epidemiology and prevention strategies for the musculoskeletal injuries in the paddle-tennis senior players. *Science & Sports* **2017**, *32*, e101-e106.

S4. Dalbøge, A.; Hansson, G.-Å.; Frost, P.; Andersen, J.H.; Heilskov-Hansen, T.; Svendsen, S.W. Upper arm elevation and repetitive shoulder movements: A general population job exposure matrix based on expert ratings and technical measurements. *Occup Environ Med* **2016**, *73*, 553-560.

S5. Andersson, S.H.; Bahr, R.; Clarsen, B.; Myklebust, G. Risk factors for overuse shoulder injuries in a mixed-sex cohort of 329 elite handball players: Previous findings could not be confirmed. *Br J Sports Med* **2018**, *52*, 1191-1198.

S6. Keener, J.D.; Skelley, N.W.; Stobbs-Cucchi, G.; Steger-May, K.; Chamberlain, A.M.; Aleem, A.W.; Brophy, R.H. Shoulder activity level and progression of degenerative cuff disease. *Journal of shoulder and elbow surgery* **2017**, *26*, 1500-1507.

S7. Klussmann, A.; Liebers, F.; Gebhardt, H.; Rieger, M.A.; Latza, U.; Steinberg, U. Risk assessment of manual handling operations at work with the key indicator method (kim-mho)—determination of criterion validity regarding the prevalence of musculoskeletal symptoms and clinical conditions within a cross-sectional study. *BMC musculoskeletal disorders* **2017**, *18*, 184.

S8. Lubiatowski, P.; Kaczmarek, P.; Cisowski, P.; Breborowicz, E.; Grygorowicz, M.; Dzianach, M.; Krupecki, T.; Laver, L.; Romanowski, L. Rotational glenohumeral adaptations are associated with shoulder pathology in professional male handball players. *Knee Surgery, Sports Traumatology, Arthroscopy* **2018**, *26*, 67-75.

S9. Saleem, M.; Tanveer, F.; Ahmad, A.; Gilani, S.A. Correlation between shoulder pain and functional disability. *Rawal Medical Journal* **2018**, *43*.

S10. Jakobsen, E.L.T.; Biering, K.; Kærgaard, A.; Dalbøge, A.; Andersen, J.H. Long-term prognosis for neck-shoulder pain and disorders: A 14-year follow-up study. *Occup Environ Med* **2018**, *75*, 90-97.

Supplementary Table S2. Above-shoulder work sensitivity analyses.

Sensitivity analysis	Studies	OR, per 1000 hours (95% CI)	Doubling dose (lifetime hours)	Supplementary Figure #
1	Dalboge 2017 men,<br>Dalboge 2017 women,<br>Svendsen 2004 men,<br>Seidler 2011 men (categorized)	1.20<br>(1.09, 1.33)	3802	1
2	Dalboge 2014 men and women<br>Svendsen 2004 men<br>Seidler 2011 men	1.15<br>(1.04, 1.26)	4959	2
3	Svendsen 2017 men and women<br>Svendsen 2004 men<br>Seidler 2011 men	1.16<br>(1.03, 1.30)	4670	3

Supplementary Figure S1. Forest plot of shoulder disease risk due to work above shoulder level (sensitivity analysis 1).

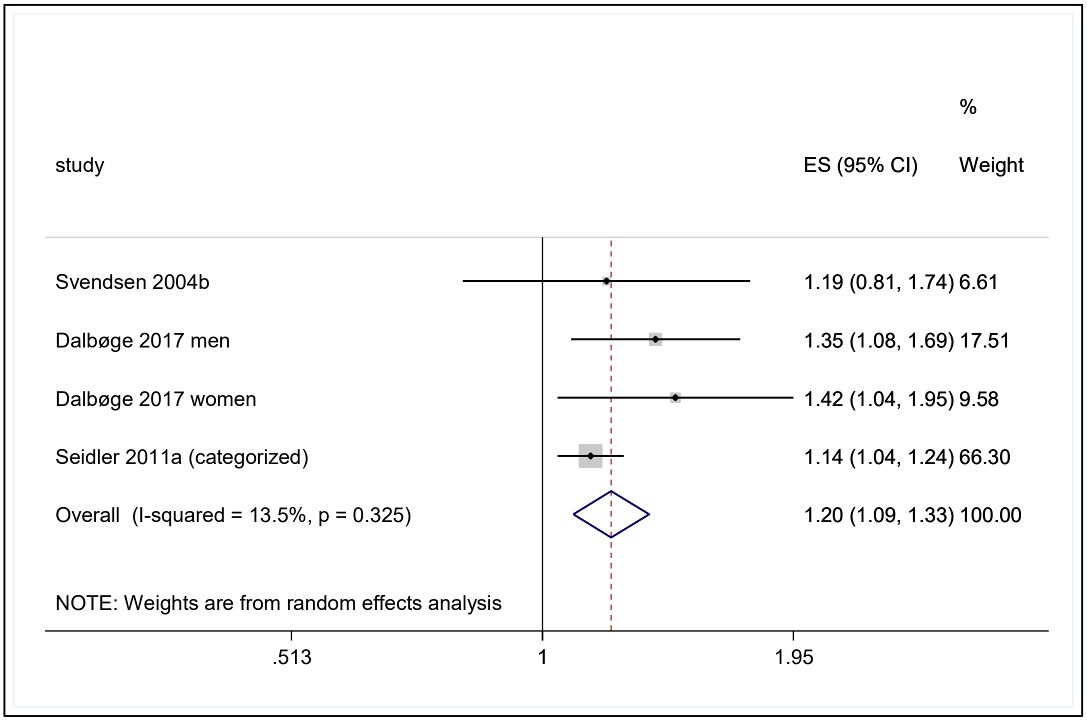

Supplementary Figure S2. Forest plot of shoulder disease risk due to work above shoulder level (sensitivity analysis 2).

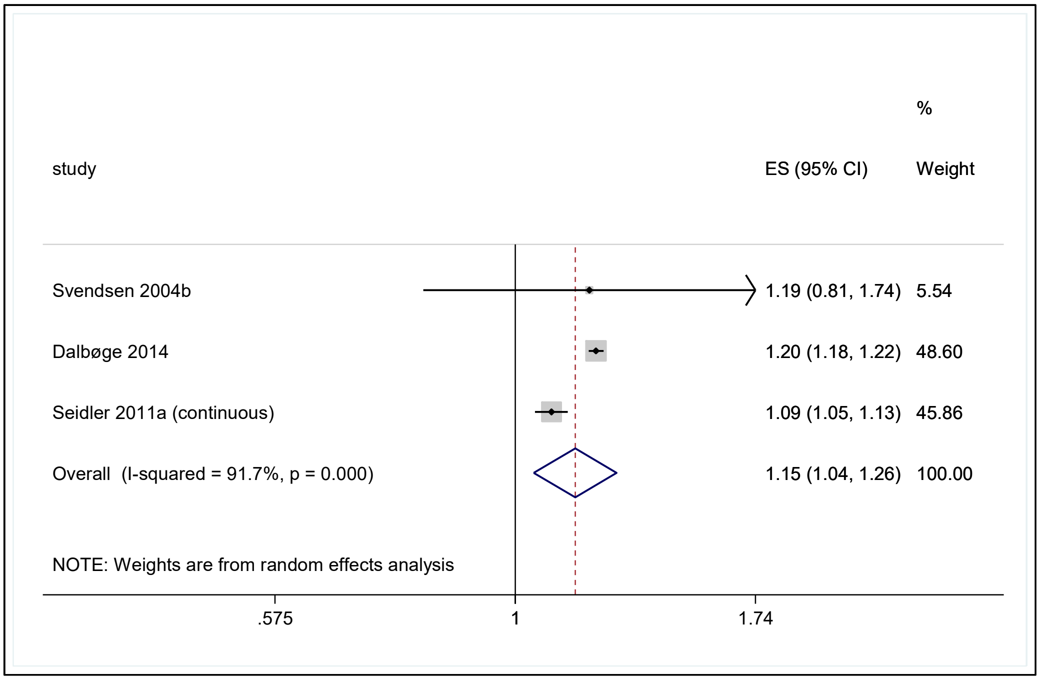

Supplementary Figure S3. Forest plot of shoulder disease risk due to work above shoulder level (sensitivity analysis 3).

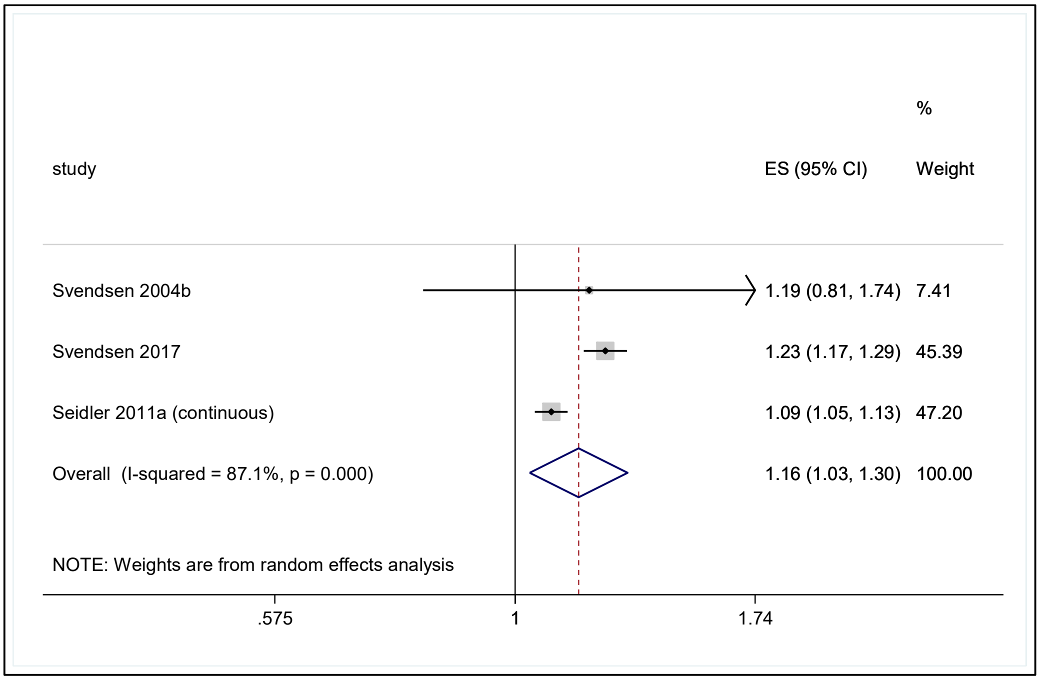

Supplement: Supplementary file 1 [file ijerph-17-01243-s001.pdf]
